# Morphologically Diverse Micro- and Macrostructures Created via Solvent Evaporation-Induced Assembly of Fluorescent Spherical Particles in the Presence of Polyethylene Glycol Derivatives

**DOI:** 10.3390/molecules26144294

**Published:** 2021-07-15

**Authors:** Mina Han, Ikue Abe, Kazunori Matsuura, Yukikazu Takeoka, Takahiro Seki

**Affiliations:** 1 Department of Chemistry Education, Kongju National University, Gongju-si 32588, Korea; 2 Department of Chemistry and Biotechnology, Graduate School of Engineering, Tottori University, Tottori 680-8552, Japan; hibari1116@gmail.com (I.A.); ma2ra-k@chem.tottori-u.ac.jp (K.M.); 3 Department of Molecular and Macromolecular Chemistry, Graduate School of Engineering, Nagoya University, Nagoya 464-8603, Japan; ytakeoka1@me.com (Y.T.); tseki@chembio.nagoya-u.ac.jp (T.S.)

**Keywords:** azobenzene-based chromophore, fluorescent micro- and macrostructures, morphological evolution, polyethylene glycol

## Abstract

The creation of fluorescent micro- and macrostructures with the desired morphologies and sizes is of considerable importance due to their intrinsic functions and performance. However, it is still challenging to modulate the morphology of fluorescent organic materials and to obtain insight into the factors governing the morphological evolution. We present a facile bottom-up approach to constructing diverse micro- and macrostructures by connecting fluorescent spherical particles (SPs), which are generated via the spherical assembly of photoisomerizable azobenzene-based propeller-shaped chromophores, only with the help of commercially available polyethylene glycol (PEG) derivatives. Without any extra additives, solvent evaporation created a slow morphological evolution of the SPs from short linear chains (with a length of a few micrometers) to larger, interconnected networks and sheet structures (ranging from tens to >100 µm) at the air–liquid interface. Their morphologies and sizes were significantly dependent on the fraction and length of the PEG. Our experimental results suggest that noncovalent interactions (such as hydrophobic forces and hydrogen bonding) between the amphiphilic PEG chains and the relatively hydrophobic SPs were weak in aqueous solutions, but play a crucial role in creating the morphologically diverse micro- and macrostructures. Moreover, short-term irradiation with visible light caused fast morphological crumpling and fluorescence switching of the obtained structures.

## 1. Introduction

The construction of fluorescent nano-, micro-, and macrostructured materials consisting of inorganic (metal and semiconducting nanoparticles (NPs)) and small organic building blocks is of enormous research interest in optoelectronics, chemistry, biomedicine, and materials science [1,2,3,4,5,6,7,8,9]. Their importance lies in the fact that artificially designed architectures with the desired morphologies and sizes may provide a variety of unexpected electronic, optical, sensing, and catalytic functions that differ from those of their small building blocks [10,11,12,13,14,15,16,17]. To produce inorganic NP-based nano- and microstructures with desired dimensions [18,19,20,21,22], top-down and bottom-up strategies have been widely developed, for example, lithographic and patterning techniques [23,24,25,26], electrospinning [27,28], the Langmuir–Blodgett technique [29,30], solvent evaporation-driven self-assembly [31,32,33], and the polymer-mediated self-assembly of NP building blocks [34,35,36,37,38,39,40,41]. Most of the bottom-up polymer-mediated NP assembly strategies require the laborious introduction of specific functional groups on NP surfaces, the addition of certain salts, pH adjustment, etc. to induce effective interfacial interactions with the functionalized polymer templates [34,35,36,37,38,39,40,41,42,43,44,45,46,47,48]. Therefore, it is important to introduce a simple and facile method to produce the desired dimensional materials.

By contrast, fluorescent organic materials (spherical, one-dimensional (1D), two-dimensional (2D), and three-dimensional (3D)) have been primarily produced based on (i) the rational design of small organic fluorophores and subsequent self-assembly through noncovalent interactions, such as hydrogen bonding, π–π stacking, van der Waals forces, and hydrophobic effects [12,13,14,15,16,17], and (ii) template-induced synthesis [49,50,51]. Thus, most studies to date have focused on how small molecular structures and their self-assembly conditions are linked to the target nano- and microstructured materials and their functions.

Han et al. recently demonstrated red fluorescent spherical particles (SPs) and 1D fibrous structures generated via the self-assembly of a new type of aggregation-induced emission enhancement (AIEE [52,53,54,55,56])—active chromophores with different terminal functional groups [57,58]. Nevertheless, it is still challenging to achieve the facile growth of such fluorescent SP building blocks into diverse micro- and macrostructured materials (from micrometer-sized chains and necklaces to macrometer-sized interconnected network structures) and to obtain insight into the determinants for governing the morphological evolution.

**Figure 1:**
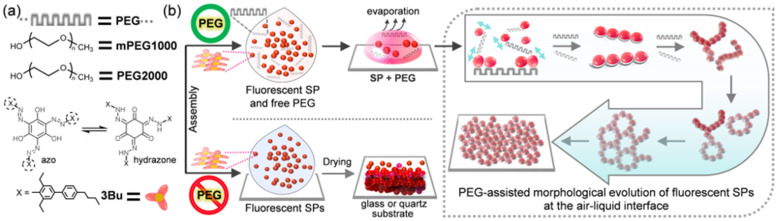
(**a**) Chemical structures. (**b**) Schematic representation of PEG-assisted morphological evolution of fluorescent spherical particles (SPs) into diverse micro- and macrostructures via solvent evaporation. Without PEG chains, Bu SPs were heaped up to form random mounds instead of interconnected networks and flat sheet structures.

Polyethylene glycol (PEG), which contains a repeating unit of –(CH_2_CH_2_O)_n_–, possesses the following characteristics: (i) It dissolves in water, as well as in commonly used organic solvents. (ii) The ethylene unit and oxygen in the PEG chain can occasionally show amphiphilic characteristics that have hydrophobicity and hydrophilicity [59,60,61,62,63]. (iii) It does not aggregate in a dilute aqueous solution [64,65]. (iv) It does not interfere with the spherical assembly of AIEE-active propeller-shaped chromophores (Bu, Figure 1). (v) It has wide-ranging chemical and biomedical applications [59,60,61,66,67,68,69]. In this study, we chose commercially available polyethylene glycol (PEG) derivatives to link SPs for these five reasons. Here, we describe a simple bottom-up approach to creating diverse micro- and macrostructures via the solvent evaporation-induced assembly of Bu SP building blocks with the help of PEG chains (Figure 1). As the PEG fraction and the PEG length increased, the SPs connected faster and generated micrometer-sized linear and branched chains. They then linked together to develop into macrometer-sized interconnected networks and sheet structures, as the solvent evaporated further. In addition, we also investigated their visible-light-triggered morphological crumpling.

## 2. Results and Discussion

### 2.1. Growth of Organic SP Building Blocks into Diverse Micro- and Macrostructures

Whereas flexible PEG does not aggregate in a dilute aqueous solution, photoisomerizable (*C*_3_-symmetric→asymmetric conformation changes) Bu has a strong tendency to assemble into fluorescent SPs [58], and the resultant SPs are well dispersed in THF-H_2_O mixed solutions. Therefore, we hypothesized that if a linear PEG chain does not interfere with the spherical assembly of Bu in a PEG:Bu binary mixed solution, the SPs and water-soluble PEGs would move independently in dilute solutions. However, slow solvent evaporation would improve the frequency of effective collision between SPs and PEG chains. As a result, the relatively hydrophobic SPs would be connected by linear PEG with amphiphilic characteristics through hydrophobic interactions [59,60,61,62,63] to evolve into larger, interconnected structures (Figure 1).

**Figure 2:**
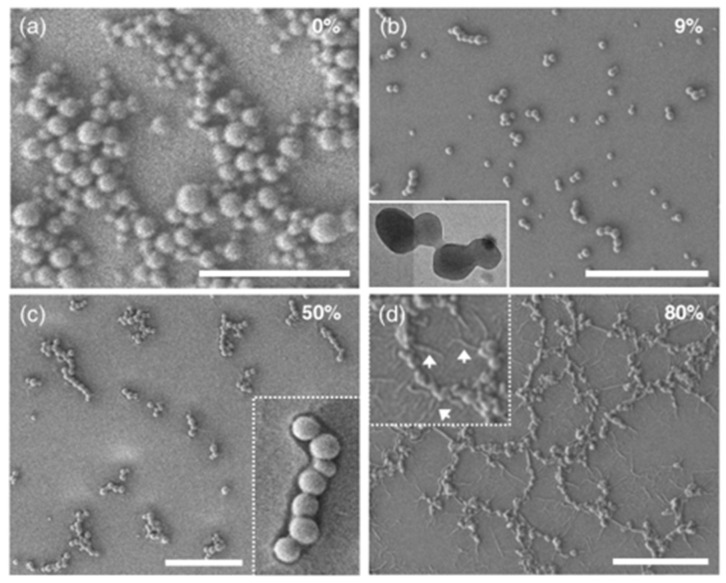
SEM and TEM images of diverse micro- and macrostructures formed at various mixing ratios (*v*/*v*) of mPEG1000:Bu (50 mg/L:50 mg/L) THF-H_2_O mixed solution. *f*_mPEG1000_ = (**a**) 0%, (**b**) 9%, (**c**) 50%, and (**d**) 80%. Scale bar: 5 μm.

To test our hypothesis, we first changed the mPEG1000 concentration (from 1 to 50 mg/L, in H_2_O) at a fixed Bu concentration (50 μM = 50 mg/L, in THF), followed by varying the mixing ratio (*v*/*v*) of mPEG1000:Bu (mPEG1000 fraction = *f*_mPEG1000_, %) and the molecular weight of PEG. According to our preliminary experimental results, the spherical assembly of Bu was not hindered by the coexistence with PEG chains and provided fluorescent SPs with diameters of ~50–500 nm. In addition, the PEG fraction had an important role in connecting SPs. For instance, in the initial stage with a small fraction of PEG (*f*_mPEG1000_ = 9%), individual SPs underwent random motion without conspicuous flocculation. However, as the solvents evaporated, the SPs very slowly connected to produce short linear chains containing ≤5 spheres (Figure 2b). Slightly longer chains and partially branched chains, which are composed of approximately ≤20 SPs, were frequently produced from a sample containing a PEG fraction of 50% (Figure 2c and Supplementary Materials Figure S1). The inset scanning electron microscopy (SEM) image in Figure 2c confirms that organic SPs forming such chains roughly retained their original spherical shape and were nested inside pea-like frames, which were presumably composed of PEG chains.

**Figure 3:**
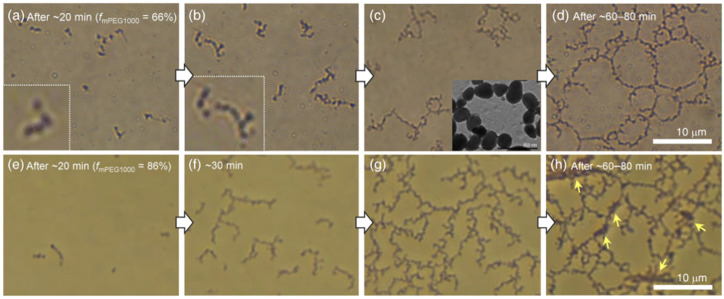
Snapshot optical microscopy (OM) images showing the morphological growth processes of mPEG1000:Bu mixed systems at the air–liquid interface. *f*_mPEG1000_ = (**a**–**d**) 66% and (**e**–**h**) 86%. (**a**,**e**) Short chains. (**b**,**f**) Relatively long and branched chains. The inset photograph in (**c**) is a TEM image of a necklace-like structure. (**d**) Interconnected structures formed by merging many necklace-like structures (see Movie S3). (**g**,**h**) Dendrimer-like structures were connected to form a giant interconnected structure. The black regions marked by the yellow arrows (**h**) correspond to densely packed SPs. The whole process took about 80–90 min under our experimental conditions (22–23 °C and 50–55% humidity).

In contrast, at higher PEG fractions, such as 66%, 80%, and 86%, longer branched chains were often observed together with interconnected networks formed by merging many necklace-like structures that were mostly 2–10 μm in size (Figure 2d and Figure 3). Notably, the inset SEM image in Figure 2d shows the existence of thread-like streaks around the network structures, suggesting that the interconnected structures were buttressed by the PEG shell. The inner SPs in the shells often turned into an oval or short rod shape, which is likely due to the long-term interactions between soft SPs and long PEG chains at the air–water interface.

### 2.2. Control Experiments: Need of PEG Chains for Morphological Growth

We conducted controlled experiments to validate the need for PEG for the morphological evolution into larger, more intricate microstructures. The importance of the presence of PEG was clearly verified at the border where solvents evaporated. In the absence of PEG, fast-moving SPs behaved independently and were heaped up to form random mounds on a hydrophilic glass substrate (Movie S1 and Figure 2a). In sharp contrast, in the presence of PEG, ready-made interconnected structures were stacked sideways to produce denser structures (Movie S2).

### 2.3. In Situ Morphological Evolution Processes at Higher PEG Fractions

To visualize the morphological evolution process, we next carried out in situ OM observations of mPEG1000:Bu binary mixed systems (*f*_mPEG1000_ = 66 and 86%) by gradually evaporating the solvents. Upon incubation for ~20 min under our experimental conditions (22–23 °C and 50–55% humidity, Figure 3a), the spheres started to slowly hook up to virtually invisible things considered to be mPEG1000, resulting in short chains. As the solvent evaporated further, the short chains linked together to form longer chains and branched chains (Figure 3b). In the case of the mPEG1000:Bu binary mixed system with *f*_mPEG1000_ = 66%, the long branched chains and round necklace-like structures were connected to one another to construct giant interconnected structures larger than 100 µm (Figure 3c,d). Importantly, once the SPs were hooked up to invisible PEG chains, the resultant intricate structures did not separate but rather floated like a single group at the air–water interface (Movie S3). These results support our earlier hypothesis that weak noncovalent interactions between the relatively hydrophobic SPs and the amphiphilic PEG chains play a crucial role in evolving into diverse micro- and macrostructures.

**Figure 4:**
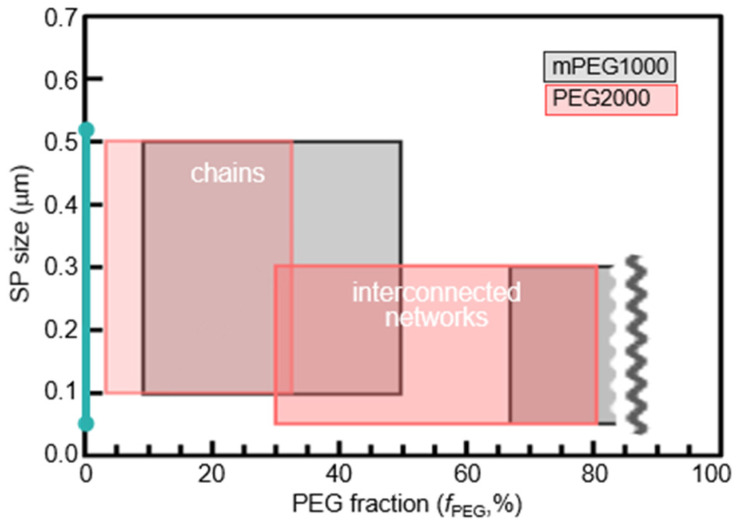
Sizes of SPs and morphologies of micro- and macrostructures formed at different PEG fractions and lengths.

**Figure 5:**
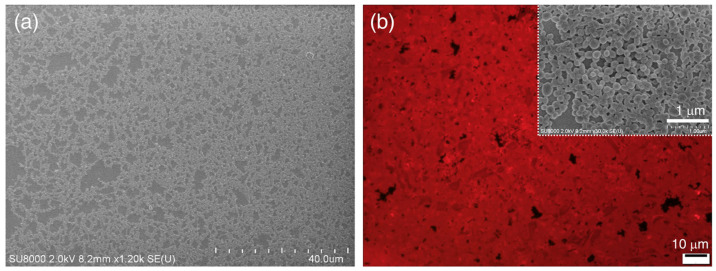
(**a**) Mesh-like network (SEM) and (**b**) red fluorescent 2D sheet (fluorescence optical microscopy (FOM)) structures obtained from the PEG2000:Bu mixed system (*f*_PEG2000_ = 80%). The inset photograph in (**b**) is a magnified SEM image of a sheet.

Moreover, when the mPEG1000 fraction increased up to 86% (Figure 3e–h) and the molecular weight of the PEG increased to 2000 (Figure 4 and Figure S2), SPs connected faster to form tortuous long chains and branched chains in the early stage, which, in turn, grew into dendrimer-like structures with many branches. As the solvent evaporated further, the large dendrimers stuck together and gradually developed into a giant mesh-like structure (Figure 5a, Figure S3). In the meantime, SPs became densely packed, as indicated by the yellow arrows in Figure 3h. Further solvent evaporation caused the mesh-like networks to be stacked horizontally on a hydrophilic glass substrate, occasionally producing a red fluorescent sheet exceeding 100 µm in size (Figure 5b).

### 2.4. UV-Vis Absorption and IR Measurements

**Figure 6:**
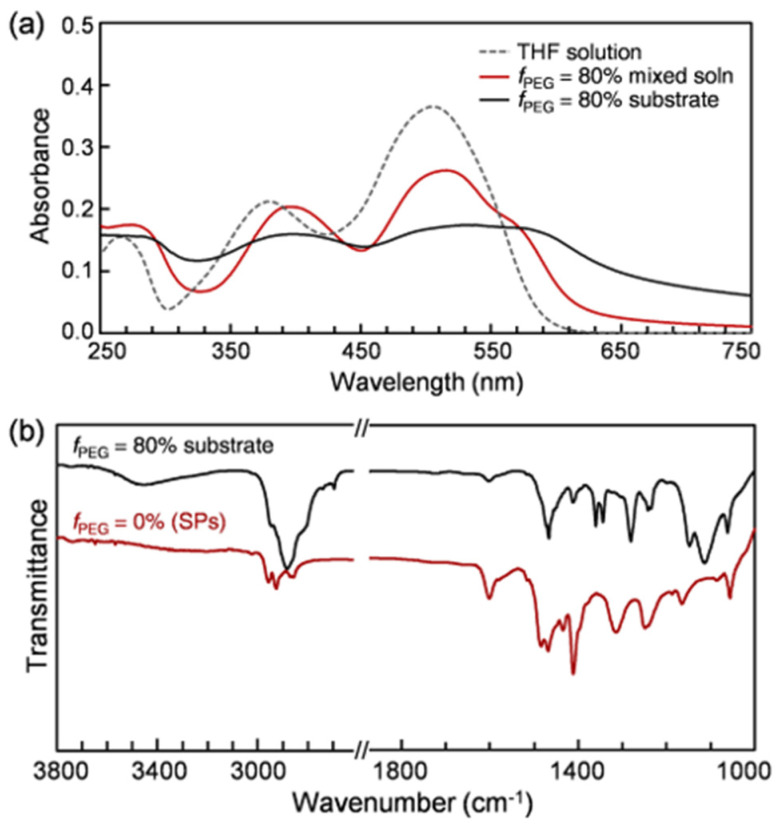
(**a**) UV-vis absorption and (**b**) FT-IR spectra.

Figure 6 shows UV-vis absorption and Fourier-transform infrared (FT-IR) spectra. A Bu dilute solution displays three characteristic absorption bands at 265, 380, and 506 nm (Figure 6a), which are likely due to the short-axis Φ–Φ* transition [70], the π–π* transition of the azobenzene unit, and the combined effect of an intramolecular proton-transfer reaction (keto-hydrazone form) and the energetic proximity of the (π,π*) and (*n*,π*) states [71,72,73,74,75,76,77], respectively. As the spherical assembly of Bu molecules and subsequent PEG-assisted morphological evolution proceeded, the three absorption bands became broader and red-shifted to >275, 396, and >515 nm, respectively.

In addition, unlike a Bu SP sample in the absence of PEG, a quite broad band in the range of 3600–3100 cm^−1^ and strong bands in the range of 2950–2800 cm^−1^ emerged in the IR spectrum for fully dried interconnected structures (Figure 6b), which are attributable to hydrogen-bonded O–H and sp^3^ C–H stretching vibrations mainly originating from PEG chains, respectively. By comparison, the phenyl-hydrogen stretching mode at 3020 cm^−1^ and the aromatic C–C stretching vibrations at 1601, 1484, and 1469 cm^−1^, which originate from Bu chromophores, considerably weakened. We interpreted these experimental results as follows. As the PEG fraction increases, compared to the terminal –OH group, the number of –(CH_2_CH_2_O)_n_– units increases enormously. Therefore, the hydrophobic ethylene units are likely to be frequently exposed to the hydrophobic parts of Bu SPs through hydrophobic interactions [59,60,61,62,63]. By contrast, the hydrophilic adjacent oxygen and –OH group seem to be mainly directed toward water and form hydrogen bonds with water molecules. Such noncovalent interactions between PEG chains and SPs are weak but not inescapable. That is, an amphiphilic PEG chain (i) acts as an important linker connecting the individual SPs in the early stage of solvent evaporation and (ii) subsequently helps the resulting shorter chains evolve into larger, interconnected micro- and macrostructures.

### 2.5. Light-Sensitive Interconnected Structures

Irradiation of AIEE-active Bu with visible light leads to changes in the UV-vis absorption spectra almost identical to those of 365 nm light irradiation (Figure S4) [58]. Notably, ^1^H NMR data measured after exposure to sunlight indicate that *C*_3_-symmetric→asymmetric conformation changes are caused by light in the region from ultraviolet to visible light (Figure S4c). Hence, we expected that if the Bu SPs were indeed nested in a pea-like PEG frame or shell with low melting points [78], the obtained diverse micro- and macrostructures would undergo discernible morphological deformation originating from light-induced conformation changes of Bu chromophores.

**Figure 7:**
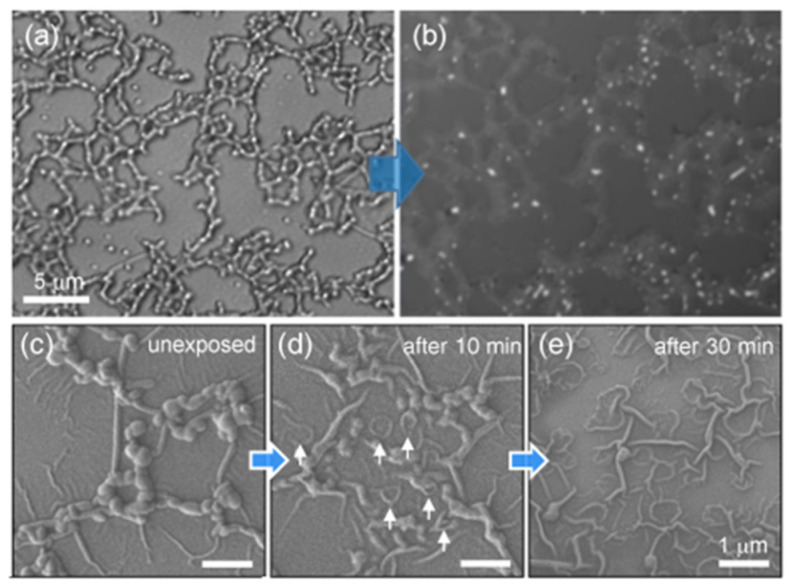
Visible-light-triggered morphological deformation of the samples obtained from the mPEG1000:Bu mixed system (*f*_m__PEG1000_ = 80%). LCM images obtained by the (**a**) first and (**b**) second measurements under light illumination (λ_ex_ = 405 nm). (**c**–**e**) SEM images showing the light-induced crumpling processes of the interconnected structures under irradiation with light at 436 nm (1–2 mW/cm^2^). Only crumpled PEG shells remained after sufficient light exposure (**e**).

To check our assumption, we exposed samples to visible light (405 and 436 nm). First, compared to the laser confocal microscopy (LCM) image obtained by the first measurement under light illumination (λ_ex_ = 405 nm), the image obtained by the second measurement revealed that the color of the chains became lighter, and the width of the chains became about two or more times wider (Figure 7a,b). That is, it was almost impossible to observe the intact interconnected network structures composed of PEG and SPs with LCM because of the light-induced morphological deformation being too fast. This was in sharp contrast to the Bu SPs, which had a very slow light response, as clearly shown in the LCM image in Figure S5.

Secondly, to lower the sphere-to-isotropic phase transition rate of SPs in the PEG shells, we chose a low-intensity blue light source (~1–2 mW/cm^2^, 436 nm). The SEM image taken after short-term irradiation with blue light revealed that the center of the particle marked by a white arrow was dented, and its rounded edge still remained (Figure 7c,d). Upon sufficient exposure to blue light for 30 min (Figure 7e), all the particles inside the PEG shell fully melted, and the round shapes disappeared completely. Eventually, only the wrinkled PEG shells remained on the glass surface.

Moreover, when exposed to green light (520–550 nm) attached to the fluorescence optical microscopy (FOM), the interconnected network structures with AIEE characteristics began to melt within one second, and their red fluorescence switched off within 3–5 s (Movie S4 and Figure S6). The light response speed of the PEG:Bu binary mixed systems was ~10 times faster than that without PEG. The fast light responses were due to two-component assembly systems [79] consisting of both PEG derivatives with low melting points and fluorescent SPs with a light-induced sphere-to-isotropic phase transition.

## 3. Conclusions

Fluorescent organic micro- and macrostructures were readily formed using the PEG-assisted assembly of soft SPs building blocks at the air–liquid interface. Our experimental results revealed that their morphologies and sizes can be readily modulated from linear chains and branched chains (with a size of a few micrometers) to giant dendrimer-like structures, interconnected networks, and sheets (ranging from tens to >100 μm in size) via slow solvent evaporation. At an early stage, the amphiphilic PEG chain served as an important linker connecting the fluorescent SPs and subsequently had the long-term interactions with the SPs to create giant interconnected structures. Eventually, the PEG shell supported the fluorescent micro- and macrostructures. In addition, fast visible-light-triggered morphological crumpling and fluorescence intensity changes were successfully substantiated through OM, FOM, LCM, SEM, and in situ OM observations. These finding will be useful for mimicking stimuli-responsive biological systems found in nature.

## 4. Materials and Methods

### 4.1. Materials

Tetrahydrofuran (THF, spectroscopic grade, Kanto Kagaku, Japan) was chosen as a good solvent to dissolve the Bu molecule. Polyethylene glycol monomethyl ether 1000 (mPEG1000, average molecular weight (Mw) of 950–1050) and polyethylene glycol 2000 (PEG2000, Mw = 1900–2100) were purchased from Tokyo Chemical Industry Co., Ltd. Bu, whose molecular weight (Mw = 1003) is almost identical to mPEG1000, was prepared according to the literature [13b]. Ultrapure water (which was purified to reach a minimum resistivity of 18.0 MΩ·cm (25 °C) using a μPure HIQ water purification system, Romax, South Korea) was used for all experiments.

### 4.2. PEG-Mediated SP Assembly into Various Microstructures

mPEG1000 H_2_O solutions in the concentration range of 1–50 mg/L were added dropwise, under mild shaking, into a Bu THF solution (50 μM = 50 mg/L), respectively. The resulting turbid suspension did not precipitate for at least 2–3 days but was well maintained until the PEG fraction (by volume) reached ~86% and over. After the suspension was aged in a volumetric flask for about 20 min, ~100 μL of the mixed suspension was carefully placed onto a clean glass or quartz substrate. To minimize unexpected side effects such as a sudden fluctuation in the solvent evaporation and the resulting change in the aggregation rate, all the experiments were conducted under the same experimental conditions (22–23 °C and 50–55% humidity).

### 4.3. Characterization

Optical microscopy (OM), fluorescence optical microscopy (FOM, λ_ex_ = 520–550 nm), and laser confocal microscopy (LCM) images were taken using an Olympus BX53 microscope and LEXT OLS4000 3D laser microscope (λ_ex_ = 405 nm) after placing a few drops of the PEG:Bu SP mixed suspension onto a clean glass or quartz substrate. The FE-SEM (field-emission scanning electron microscopy: HITACHI SU8020 and TESKAN-MIRA3-LM) samples were coated with an approximately 5–10 nm-thick platinum layer using a Cressington 108 auto sputter coater, Ted Pella, Inc. The transmission electron microscopy (TEM) was performed at 120 kV using a JEOL JEM-1400 Plus. UV-vis absorption and fluorescence spectra were recorded using a Shimadzu UV-2600 UV-vis spectrophotometer and a Horiba FluoroMax-4 spectrofluorometer, respectively. Fourier-transform infrared (FT-IR) spectra were recorded on a PerkinElmer (spectrum 100) spectrometer. Samples were exposed to light (Tokina Supercure-204S, generated by a combination of Toshiba color filters) to investigate their light response.

## Data Availability

The data are included within the manuscript and Supplementary Materials.

## Supplementary Materials

The following are available online. Figure S1: SEM image of the sample obtained from the mPEG1000:Bu mixed system (f_mPEG1000_ = 50%), Figure S2: TEM and SEM images showing various microstructures formed by connecting the SPs through PEG2000, Figure S3: SEM images of mesh-like structures, Figure S4: ^1^H NMR, absorption, and fluorescence spectra, Figure S5: LCM image of relatively photostable Bu spheres, Figure S6: Visible-light-triggered morphological deformation and rapid fluorescence intensity response, Movie S1: A control experiment (mp4), Movie S2: In the presence of PEG, ready-made interconnected structures were stacked sideways (mp4), Movie S3: In situ OM observation of a giant interconnected necklace structure (mp4), Movie S4: Fast light responses: In situ FOM of mesh-like structures observed during visible light irradiation for 5 s (mp4).

## Sample Availability

Samples of Bu are available from the corresponding author.
