# Supplementary material for: Morphologically Diverse Micro- and Macrostructures Created via Solvent Evaporation-Induced Assembly of Fluorescent Spherical Particles in the Presence of Polyethylene Glycol Derivatives
Source: Molecules. 2021 Jul 15;26(14):4294. doi: 10.3390/molecules26144294 (PMC8304015; doi:10.3390/molecules26144294)
Supplement: Supplementary file 1 [file molecules-26-04294-s001.zip › 2_SM-Han.pdf]

Supplementary Materials (SM)

Morphologically Diverse Micro- and Macrostructures Created via Solvent Evaporation-Induced Assembly of Fluorescent Spherical Particles in the Presence of Polyethylene Glycol Derivatives

Mina Han, Ikue Abe, Kazunori Matsuura, Yukikazu Takeoka, and Takahiro Seki

Contents

Figure S1. SEM image of the sample obtained from the mPEG1000:Bu mixed system (f_{mPEG1000} = 50%)

Figure S2. TEM and SEM images showing various microstructures formed by connecting the SPs through PEG2000

Figure S3. SEM images of mesh-like structures

Figure S4. ^{1}H NMR, absorption and fluorescence spectra

Figure S5. LCM image of relatively photostable Bu spheres

Figure S6. Visible-light-triggered morphological deformation and rapid fluorescence intensity response

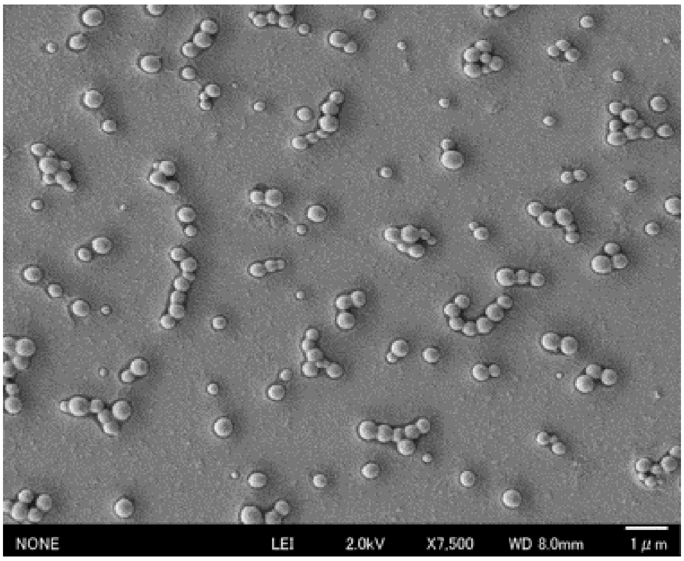

Figure S1. SEM image of the sample obtained from the mPEG1000:Bu mixed system (f_{mPEG1000}= 50%).

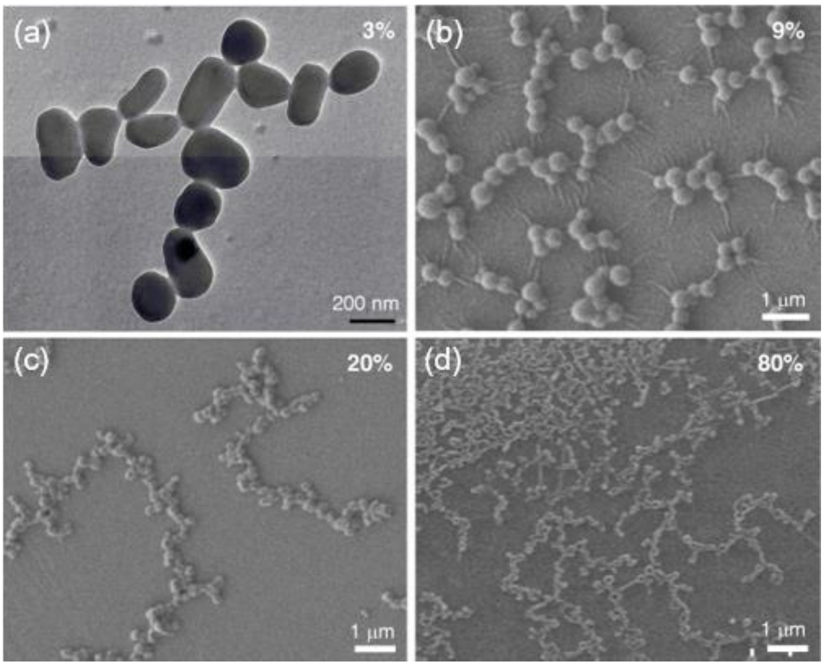

Figure S2. TEM and SEM images showing various microstructures formed by connecting the SPs through PEG2000. f_{PEG2000} = (a) 3%, (b) 9%, (c) 20%, and (d) 80%.

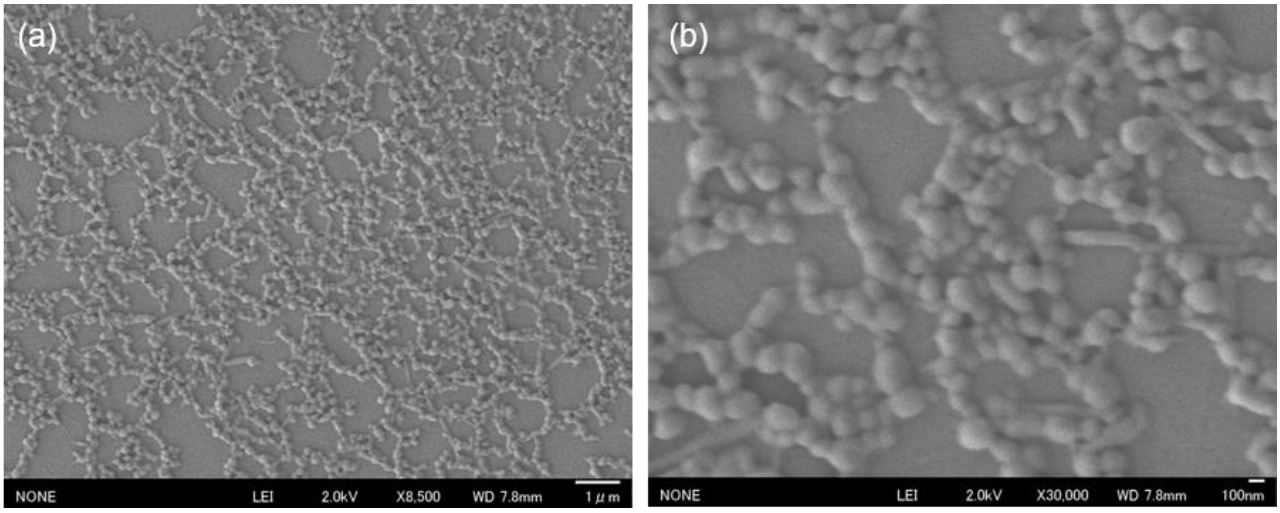

Figure S3. Mesh-like structures obtained from the mPEG1000:Bu mixed system ($f_{mPEG1000}$ = 86%). (b) Magnified SEM image.

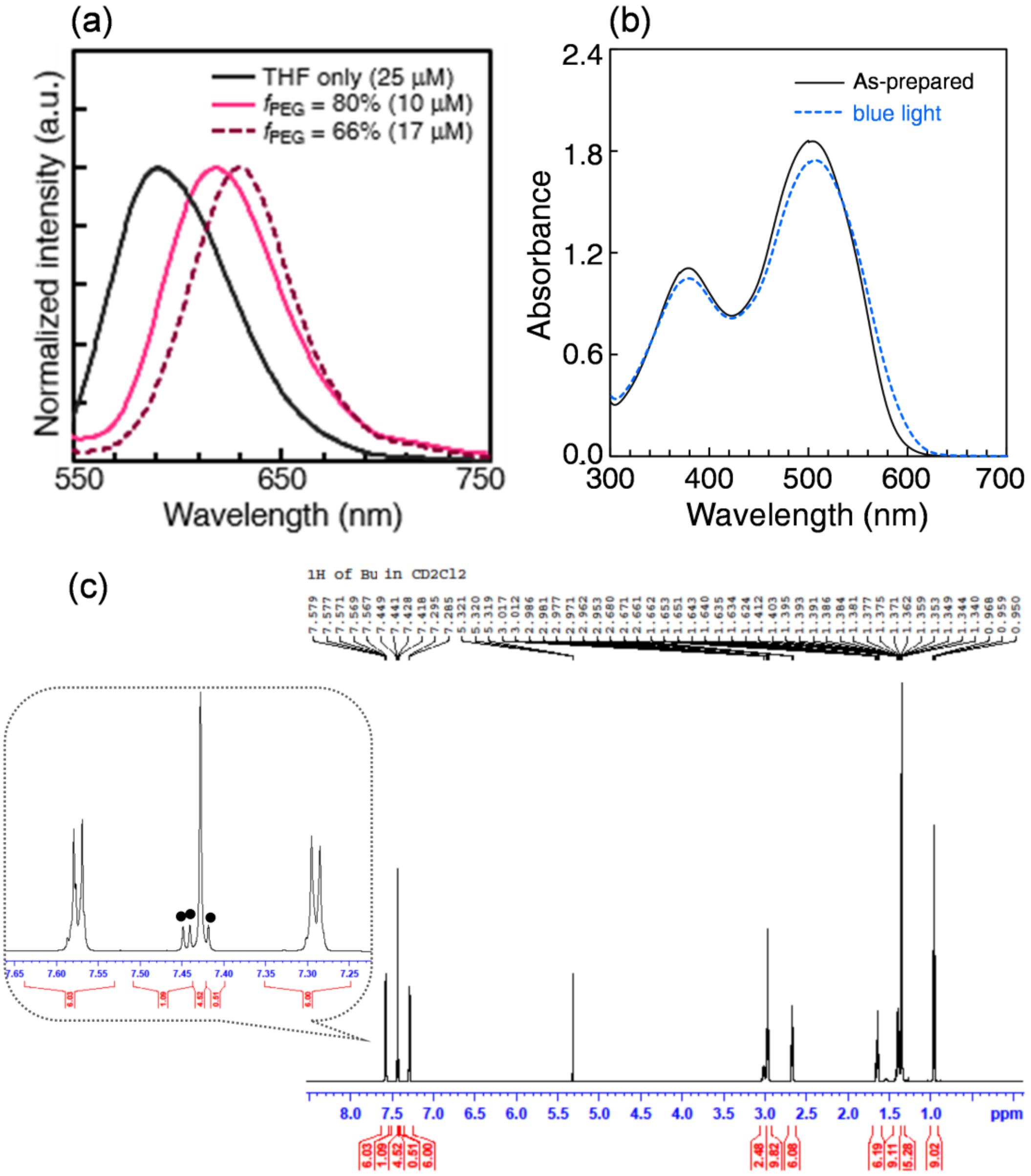

Figure S4. (a) Fluorescence spectra before and after the formation of the Bu SPs by the addition of the mPEG1000 H_2O solution to the Bu THF solution. The obtained sample showed a fluorescence quantum yield as high as ~1.9%, which was comparable to that of SPs without PEG. (b) Absorption spectral changes of Bu THF solution. (c) ^{1}H NMR data taken after exposing the Bu sample to sunlight (in CD_2Cl_2). Three singlet peaks marked with filled circles appeared upon exposure to room light.

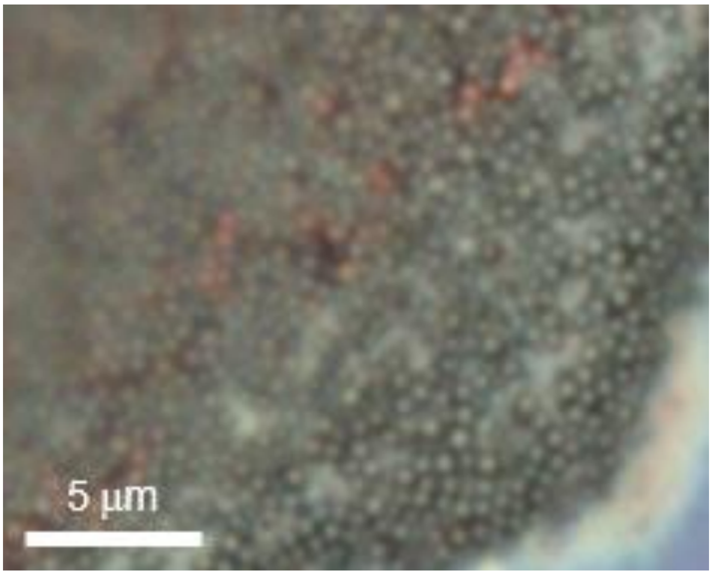

Figure S5. LCM image of relatively photostable Bu spheres. The round shape remained intact even after several LCM measurements.

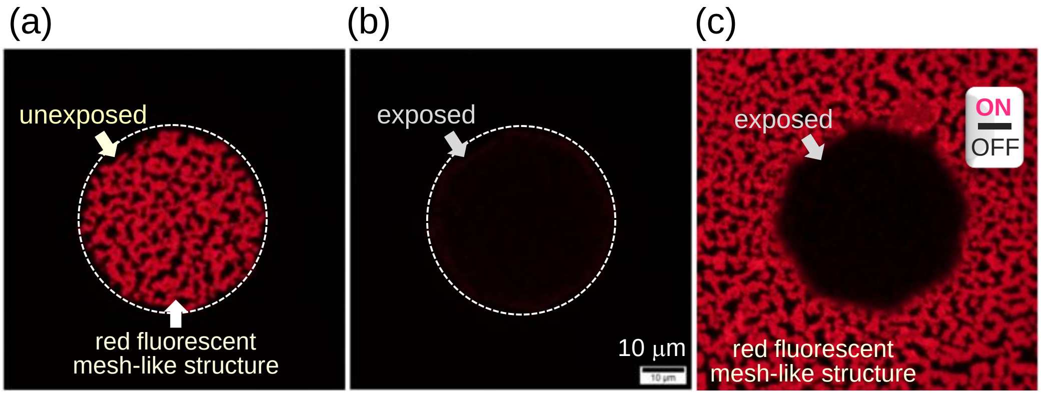

Figure S6. (a-c) FOM images of interconnected network structures taken before and after irradiation with green light (attached to FOM) for 5 seconds (λ_{ex} = 520–550 nm). The dark parts in Figure S6b-c correspond to the light-induced melting of network structures.
